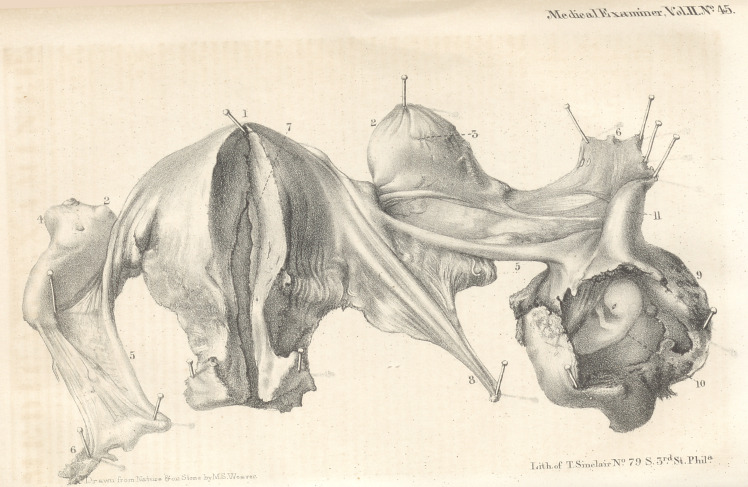# Transactions of the Pathological Society of Philadelphia

**Published:** 1839-11-09

**Authors:** 


／MEDICAL EXAMINER.

DEVOTED TO MEDICINE, SURGERY, AND THE COLLATERAL SCIENCES.

No. 45.] PHILADELPHIA, SATURDAY, NOVEMBER 9, 1839. [Vol. II.

### TRANSACTIONS OF THE PATHOLOGICAL SOCIETY OF PHILADELPHIA.

*October 14th*, 1839.

The President, Dr. Gerhard, in the Chair.

*Case of Extra-Uterine Pregnancy.* By Charles D. Meigs, M. D., Physician to the Lying-in Department of the Pennsylvania Hospital, Lecturer on Midwifery, &c. [With a lithographic engraving.]

Dr. Meigs presented a specimen of *Fallopian Pregnancy*, and furnished the following history of the case:

Mrs. ——, aged thirty-two years, had suffered from an attack of intermittent fever, about the middle of September, 1839. The intermittent appears to have been completely cured by the care of her physician, Dr. Bicknell, of West Philadelphia. Mrs. —— had four children, the youngest of whom was less than two years old. Her health and spirits were good. On Saturday morning, October 6th, 1839, she was heard singing and playing with her children, with whom she was alone, in an apartment down stairs. Shortly afterwards, between six and seven o'clock, on the morning above mentioned, she was heard groaning very heavily, and walking up stairs. Upon entering her chamber, she was seen to be alarmingly ill, with pain which affected the whole of the right front of the body, from the iliac region to the top of the thorax. Dr. Bicknell saw her shortly after the attack, and found her in very great pain, with a pulse of 140 per minute, affected with vomiting, and having a tympanitic abdomen. The remedies employed by Dr. Bicknell appeared to have some effect in appeasing the distress of the patient; and she was so far relieved, that when he left her, at 8 o'clock on Sunday morning, the pulse was beating at 120 per minute, and the pain and restlessness were considerably diminished. Upon returning, however, shortly afterwards, to the house, the symptoms were found to be aggravated, and a messenger was sent to invite me to see the lady.

I arrived at about half past two o'clock, P. M., on Sunday, and having had no intimation of the nature of her attack, I concluded it to be a case of labour, and as soon as I saw her, thought she was sinking under the effects of uterine hæmorrhage; her appearance being that of a person bleeding too much. I approached her for the purpose of removing the pillows, with which her shoulders seemed too much raised. But, upon asking whether she had lost a great quantity of blood, I was surprised to learn that she had not been attacked with hæmorrhage. I was very much disconcerted by this information, because I found that the diagnosis must be difficult in view of the exsanguinous colour, the hurried, broken respiration, the sighing, and the extreme exhaustion of the sick lady, which had led me too hastily to suppose she was flooding. Upon examining the pulse, I found it not very small,—but the effort of the heart was evidently great—the systole being sudden and frequent, but without communicating to the pulse at the wrist any firmness or strength.

The tongue was clean and moist; the skin, where exposed, was cool—under the bed-clothes the heat was considerable, with perspiration. The abdomen was highly sensible upon pressure made on any point of its surface, and the distension so great, that it was as large as that of a woman at the sixth month of gestation; it was *excessively* tense, and highly sonorous upon percussion. The patient had singultus, eructations, and, occasionally, a disposition to vomit. She announced herself to be dying, and was fully convinced that no relief could be extended to her. In about three-quarters of an hour after my arrival, she requested to be raised a little with pillows. This was done, but she became immediately faint, and, shortly afterwards, expired, without the least struggle.

I was much at a loss to account for the state of this lady, seeing that she had been in very good health only some thirty-four hours before her decease.

Notwithstanding her bowels had been moved by the remedies employed by Dr. B., I was very much inclined to think that an intussusception of the intestines, or some great obstruction of the bowel, could alone account for the speedy death which had overtaken her. I even began to feel some degree of confidence in such a view of the case; but could not avoid recurring again to the striking resemblance which her appearance bore to that of persons who had sunk under hæmorrhage.

On Monday, the 8th, at 12 o'clock, the body was examined, there being present with me, Dr. Bicknell, a gentleman a friend of the family, and a female.

The abdomen was much distended and sonorous when percussed; upon laying it open by a crucial incision, a great quantity of bloody water was removed, probably more than a pint. Next I took out between thirty and forty ounces of coagula, which not only filled the pelvis, but were found effused high up in the abdomen. The intestines were greatly distended with gas, the peritoneal coat of the bowels was highly coloured by inflammation, but I discovered no signs of any effusion of coagulating lymph, nor were there any flocks of lymph or albumen floating in the bloody serum which was contained in the belly. No adhesions were discovered. After removing the coagula, the seat of the disease was perceived; the parts were removed, and a most faithful drawing, by

Mr. Weaver, which accompanies this paper, will enable the reader to understand the nature of the accident by which the patient was so suddenly deprived of life.

The description of the drawing is in the words of Dr. GODDARD, to whom the Society is indebted for the admirable preparation with which he is about to enrich their collection.

*Description of a case of Tubal Pregnancy, as it appeared on dissection.* By P. B. GODDARD, M. D.

*Uterus.*—The uterus (fig. 1, see plate) was of a natural colour, and the size was that of a woman who had previously borne children. It was lined throughout by a false membrane, rather more than a line in thickness, (7,) extending from the top of the fundus to the intersection of the neck and body, where it tapered off to a thin edge. At this point a gelatinous mass was found, which extended to the os-tincæ, and protruded slightly from it. This little mass was perfectly transparent, very tenacious, and of the colour of Madeira wine. When immersed in alcohol, it became opaque, and coagulated into firm, interlaced fibres.

*Ovaria.*—The right ovarium had projecting from its surface a large corpus luteum, (4,) and was marked by several cicatrices. The left was marked by one cicatrix, (3.)

*Fallopian tubes.*—The right fallopian tube was normal, but the left was dilated from the root of the fimbriated extremity half way to the uterus. It had evidently been dilated to the diameter of half an inch, and its structure had separated by distention at the lower part, thus ceasing to be the envelope of the ovum.

*Ovum.*—The ovum consisted of a fœtus of about six (?) weeks, with the amnion and chorion perfect, the chorion being covered by a false caduca, varying in thickness from two lines to five. This had ruptured at the anterior part, leaving a lacerated opening, about three-fourths of an inch in length, from which the blood found in the peritoneal cavity had escaped.* The tube adhered to the left ovarium by numerous fibres, and was enveloped in a firm coagulum, rendering it impossible to distinguish it from an ovarian pregnancy, until it was carefully dissected.

*References to the plate.*

1. Uterus.
2, 2. Ovaria.
3. Cicatrix.
4. Corpus luteum.
5, 5. Fallopian tubes.
6, 6. Fimbriated extremities of do.
7. Decidua.
8. Round ligament.
9. Ovum.
10. Fœtus.
11. Dilated portion of fallopian tube.

*Remarks by Dr. Meigs.*

With the permission of the Society, I shall venture to offer a few remarks suggested by the circumstances of this case:

1st. The wound occasioned by the rupture of the tube, ought not to be considered as alone sufficient to account for the sudden destruction of a healthy adult. Much more extensive wounds of the gestative organs, as in the Cæsarean operation, and in lacerations in labour, do not necessarily cause the death of the patient; and we know that the removal of the ovaria, so frequently practised upon some of the inferior animals, is rarely followed by either extensive inflammation or death.

2d. The loss of forty or fifty ounces of blood, especially when the effusion requires many hours to effect it, is not sufficient to cause the death of an adult.

3d. Mere active, red inflammation of the peritoneal coat of the intestine, does not so speedily cause the death of the patient. Several days must elapse, and the inflammation should come to one of its terminations, as, for example, that of inflammatory effusion into the sac, before death closes the scene.

4th. I have already remarked upon the extreme and painful tension of the abdomen in this case, and I venture to suggest to the Society a consideration of the violent perturbation introduced into several important functions by this tympany, which so generally accompanies the peritoneal forms of fever.

(1.) The peritoneal coat of the bowel being inflamed, its muscular coat becomes quiescent or inactive, and permits the tube to be distended by the gases that are extricated in the stomach or intestines. Muscles of volition refuse to act in obedience to the will, when their contractions cause pain in the parts moved by them. A muscle lying under an extensively inflamed portion of skin loses its voluntary contractility, on account of the pain its motion produces, and even the extensors of the leg will not act if the knee-joint is acutely inflamed and sensible. Examples of this pathological sympathy are so numerous and obvious, that they need not be further cited here. It appears to me that the same pathological sympathy may be presumed to exist in the involuntary muscles.

It is rarely that we meet with a case of peritonitis, whether puerperal or not, without discovering an early proneness to tympanitic distention of the abdomen. The distention takes place almost as soon as the pain and soreness begin to be felt. Can any reason be assigned for this symptom other than the suspension of the peristaltic power caused by the inflamed state of the peritoneal coat of the bowels. The serous coat being inflamed, the muscular coat loses its contractility, and passively yields to the distending force of the gases extricated within the tube, which inflate it to its utmost limit of expansion. I do not know whether the inflammation of the serous coat is early propagated into the muscular tissue which lies under it; I presume it is not immediately.

(2.) The violent distention of the alimentary tube, which is the direct cause of much pain in the bowel itself, produces upon the whole

* This was stated by Dr. Meigs to be from thirty to forty ounces.

Drawn from Nature & on Stone by M.S. Weaver.

Lith. of T. Sinclair No. 79 S. 3rd St. Phila.

nervous apparatus of the belly the effect of a great shock; and the perturbation extends to various organs, so as to exert the most pernicious influence upon the patient's vital forces.

(3.) The diaphragm does not descend freely upon the inflamed and distended mass contained within the abdomen, not only because it meets with a great physical resistance, but because it gives rise to violent pain by its pressure in descending. Hence, in these cases, the respiration becomes short and hurried, being often thirty-five or forty per minute, and simulating the pleuritic respiration. This phenomenon cannot co-exist with a normal performance of the chemical functions of the lungs; therefore, in bad cases, the patient soon becomes pale, assumes a peculiarly haggard appearance, and a slight degree of asphyxia becomes discoverable in the bluish-pale tint of the face,—the eyes grow dim,—the mind languishes, or is calm, at least,—a mortal coldness and blueness seize upon the extremities of the hands and feet, because the calorific and decarbonizing functions of the lungs very early give way under the circumstances in question.

As this is not a proper place to extend these remarks, I shall beg leave to refer for my views more *in extenso*, to page 354 of the Phil. Pract. of Mid., published here two years since.

The rapid dissolution of the lady whose case I have related, and the appearances discovered upon the dissection, confirm me in the justness of the pathological views I have expressed, and which I have long entertained, as to the pernicious tendency of a tympany accompanying peritonitis. If they are well founded, it becomes a most important question, whether we can safely undertake the treatment of peritoneal fevers without the aid of cathartic or aperient medicines, which alone are capable of exciting the peristaltic fibres while invested by an inflamed serous membrane. The carminative doses sometimes resorted to in order to get rid of the inconveniences of the tympany, are powerless in tympany from peritoneal inflammation. It is quite certain, I think, and I have fully expressed that opinion, that a bold use of the lancet is *the remedy* for such disorders. Yet the treatment of them, in order to be successful, requires the aid at least of aperients, repeated sufficiently often to keep down the dangerous disturbance of the economy, which cannot but follow an over-distended bowel in peritoneal fevers. In regard to the particular case of Mrs. ———, it is clear that the injury was mortal from the moment of the tube's rupture. I have endeavoured to explain the rapidity of the case by calling your attention to the perturbing influence of the extraordinary and extensive tympany.

—

*8th November*, 1839.

The President, Dr. Gerhard, in the chair.

Dr. J. Forsyth Meigs exhibited a specimen of *Fibrinous Tumour of the Breast*, and read the following account of the case:

Elizabeth M——, a coloured girl, aged fifteen years, entered the Pennsylvania Hospital on the 25th of October, 1839, for a tumour, occupying the left breast. She is remarkably well developed for her age, and states that she has always been of a healthy and vigorous constitution. Her parents are still living, and in good health. She does not know that any of her relatives have suffered from a disease similar to hers. She first menstruated somewhat more than two years ago, and has been perfectly regular since. About two years before her coming to the hospital, while engaged as a domestic in a healthy part of the country, her attention was for the first time called to a hard, circumscribed, moveable tumour, of about the size then of a hazel-nut, occupying the upper, outer part of the breast. The tumour has continued gradually to increase in size up to the present time, without causing any interruption to her usual good health, and producing no inconvenience whatever, excepting slight dull pain now and then, lasting but for a short period. She can assign no cause whatever for its production.

*November 5th.*—The tumour is somewhat oblong in shape, with its longest diameter vertical; consisting apparently of two substances, one rather firm and unyielding, feeling smooth to the touch—the other consisting of a number of rounded bodies projecting from the first, elastic, with a sense of obscure fluctuation. The whole mass is perfectly moveable under the skin, without any connection apparently with either the integument or muscle. There is no pain even upon handling it roughly, and the nipple is free and not retracted; two veins of much larger size than any on the opposite breast may be seen running across the tumour. The mammary gland may be felt in part beneath the tumour, of less size than the opposite one. There is no enlargement of the glands of the axilla.

On the 6th of November Dr. Harris removed the tumour, in connection with the mammary gland. The hæmorrhage was considerable, though no very large arteries were divided. The number of ligatures amounted to nine.

*Examination of the tumour.*—The tumour, when regarded by the edges, is of an irregular oblong shape. The posterior face, that applied to the pectoral muscle, is an irregular plane, rendered uneven by the projection of a number of rounded lobules, varying in size from a quarter to half an inch. The anterior surface is of a convex shape, owing to the lobules being much larger, and also from the tumour being much the thickest in the centre. The dimensions are as follows:—greatest diameter, that which presented vertically, four inches; transverse, three and a half inches. The greatest thickness is in the centre, two and a half inches; from this it slopes towards the edge, where it varies from a quarter to half an inch. There are three entire sacs forming coverings to the tumour. The most external is formed of a thin, easily torn, cellular tissue; this is readily dissected from the surface of the tumour, and passes from the top of one projection to that of the next, dipping but a very short distance into the sulcus between the two lobules.

The second tunic is also formed of cellular tissue, much more condensed, and more closely applied than the first, passing like that, also, from one projection to the next, and reflected but a short distance into the sulci. This appears to hold the lobules together, giving them considerable support. The third covering is of a cellulo-fibrous texture, somewhat thin and attenuated upon the exterior, becoming more dense, with greater evolution of the fibrous structure, as we proceed towards the interior of the tumour. This membrane dips down into the sulci to various degrees of depth, and contains within it the proper, peculiar matter of the disease.

Upon making an incision through the central part, it presents the appearance of a number of lobes, of various shape and size, fitted closely together, and connected by short laminæ of cellular substance. These lobes appear to be again divisible into lobules, formed of the proper structure of the tumour developed in the meshes of a fine cellular tissue. The cut surface resembles more closely a section of the pancreas than any thing else to which I can compare it: it has a dull-white, shining aspect, and when the finger is passed over it, gives an impression of irregularity, owing to the division into lobes and lobules, while each separate lobule feels smooth and polished. It is of moderately firm consistence, cuts without yielding under the scalpel, and does not give a crying sound.

By careful dissection, it may now readily be seen that each projecting lobule forms the summit of a body of a pedunculated form, the bases of which, in the smaller bodies, are directed towards, and spring from the larger,—while in the case of the latter they can be traced as radiating from a common centre, placed in the posterior part of the tumour. Each pedunculated body consists of the proper structure of the tumour enclosed in the cellulo-fibrous sac, adhering to each other laterally by short bands of cellular tissue, readily divided by the back of the knife. The membrane can be traced to the bottom of each sulcus, when, as we reach the origin of the body under examination, it may be seen reflected across to the one adjoining, thus preventing any further prosecution of the dissection in this direction without cutting through this sac.

*Remarks.*—The most interesting question with regard to the tumour in question, is the following: Is it, or is it not liable to be reproduced? To determine this satisfactorily I have consulted various authorities, and shall proceed to give the result of my inquiries.

Mr. Abernethy, in his Surgical Observations, describes a tumour very nearly resembling the one under consideration. This he calls *pancreatic sarcoma*, and says: "This new-formed substance is made up of irregularly-shaped masses, in colour, texture, and size, resembling the larger masses which compose the pancreas. They appear also to be connected with each other, like the portions of that gland, by a fibrous substance of a looser texture." He states that he has frequently seen the axillary glands enlarged in connection with this kind of tumour, and yet even after this, there has been no return of the complaint after excision. Sir A. Cooper describes, under the term of simple chronic tumour of the breast, a disease answering very closely to the appearance of the above. He thus describes it: "Upon dissection, the swelling is found to be composed of a number of lobes connected together by a condensed cellular tissue, and which appear as enlargements of the lobes of the mammary gland. These lobes are composed of smaller, which, by maceration, may be separated. The appearance of the disease, when cut into, is that of sweet-bread, that is, lobulated in every part, or composed of large lobes, which are divisible into smaller." He considers this disease not to be malignant, and mentions that it attacks the young and apparently healthy. In the last number of the new Dictionnaire de Médecine, M. Velpeau, in the article upon the mammæ, describes, under the head of fibrinous tumours, one very similar to the above. He says: "I include under this title, (fibrinous tumours,) masses variable in regard to their colour, consistence, and the manner in which they are connected with the surrounding tissues, but which possess the common characters of being inclosed in one or more cysts, of acting amongst the tissues as foreign bodies, and of resembling none of the natural organic elements of the body. The volume of these tumours varies from that of a small nut to that of the head; they are usually studded with projections, irregular, and elastic or fungoid. When cut into they appear somewhat like a lymphatic ganglion, distended or enormously hypertrophied, lobulated, friable, breaking under the pressure of the finger; in other cases they resemble exactly old fibrinous concretions which have become organized. They are sometimes sufficiently firm and homogeneous to resemble the texture of scirrhus, or fibrous productions." M. V. states that these tumours are met with more frequently among young girls and unmarried women than any others. He classes them among the benign tumours, and advises extirpation, having as yet seen no case of return after the operation.

I have consulted also the works of Boyer and Dupuytren, but can find no description resembling exactly the one under consideration.

Mr. Carswell says that when the heterologue deposit "assumes a regular, lobulated arrangement, so as to represent an appearance similar to a section of the pancreas, it forms what was called by Mr. Abernethy the pancreatic sarcoma." This he places in the genus Carcinoma, and states that it is liable to be reproduced.

Dr. Hodgkin's description of the formation of malignant tumours coincides almost precisely with the anatomical arrangement found in this disease. He says that they consist generally of pedunculated bodies having a common origin for their peduncles, and not connected by their sides. His theory with regard to their formation is as follows:—He supposes them to consist of a cyst, containing in its cavity packets of pedicles, and bodies also inclosed in cysts. In those tumours where the internal growth is most active, the contained cysts enlarge more rapidly than the containing, thus producing its rupture, and, advancing rapidly through the opening, in this way increase the size of the tumour.

Having thus quoted the opinion of several writers with regard to the malignancy of tumours like that which is the subject of the above description, I find that Velpeau, Sir A. Cooper, and Mr. Abernethy, all declare the disease not likely to be reproduced, as also Drs. Harris, Randolph, and Norris, who were present at the operation, and who at once declared the tumour to be non-malignant; while opposed to this opinion are Mr. Carswell and Mr. Hodgkin.

---

*Philadelphia, November 8th,* 1839.

To the Editors of the Medical Examiner.

GENTLEMEN,—Having received, through the kindness of a friend, the "Ninth Report of the Ophthalmic Hospital at Canton, for 1839," by the Rev. P. Parker, M. D., I hope that the subjoined list of diseases treated, and abstract of some of the cases, may not be without interest to your readers.

Yours, respectfully,
A. DENMAN CHALONER.

Since the last report, the growing confidence reposed by the people in the skill of the *foreign* surgeon has been strongly displayed in the degree of readiness with which they submit to painful operations, and even to the loss of limbs, although this is so greatly opposed to their prejudices and principles.

A Chinese female (the first, so far as we know, at least in modern times) has submitted to an amputation of her right arm; and four others have undergone extirpation of their breasts, on account of cancerous disease in an advanced stage.

During the months of July, August, and September, the Hospital was closed, and under repair, and that at Macao meanwhile opened.

The patients that have been admitted during the term, are 505; the aggregate, since the opening of the institution, is 6300.

The following is a tabular statement of the numbers of each disease that have come under observation during the past term.

*Diseases of the Eye.*

| | |
|---|---|
| Granulations, | 18 |
| Ectropium | 1 |
| Entropium, | 46 |
| Trichiasis, | 1 |
| Lippitudo, | 15 |
| Xeroma, | 1 |
| Hordeolum, | 1 |
| Excrescences of the lids, | 1 |
| Quivering lids, | 1 |
| Paralysis of muscles, | 1 |
| Obstruction of nasal duct, | 3 |
| Disease of Caruncula Lachrymalis, | 2 |
| Ophthalmia, Acute, | 21 |
| " Chronic, | 84 |
| " Purulent, | 3 |
| " Scrofulous, | 2 |
| Ophthalmitis, | 1 |
| Pterygia, | 22 |
| Acute inflammation of the cornea, | 1 |
| Nebulæ, | 44 |
| Ulceration of cornea, | 2 |
| Opacity of cornea, | 3 |
| Leucoma, | 1 |
| Staphyloma, | 20 |
| Iritis, Chronic, | 3 |
| Synechia, Anterior | 5 |
| " Posterior, | 5 |
| Closed pupil, | 2 |
| Choroiditis, | 1 |
| Cataracts, | 44 |
| Glaucoma, | 2 |
| Musculi volitantes, | 2 |
| Myosis, | 3 |
| Amaurosis, | 4 |
| " Partial, | 2 |
| Myopia, | 2 |
| Day blindness, | 1 |
| Night blindness, | 1 |
| Fungus hæmatodes, | 1 |
| Loss of one eye, | 22 |
| " of both eyes, | 12 |

*Miscellaneous Diseases.*

INFLAMMATORY DISORDERS.

| | |
|---|---|
| Rheumatism, | 6 |
| Thrush, | 2 |
| Abscesses, | 5 |
| Arthritis, | 2 |
| Fistulous mammæ, | 1 |
| Fistula, (in ano,) | 1 |
| Ulcers, (chiefly of lower extremities,) | 3 |
| Ulceration of fauces, | 1 |
| Inflammation of fauces, | 1 |

*Constitutional Diseases.*

| | |
|---|---|
| Ascites, | 2 |
| Anasarca, | 6 |
| Opium mania, | 8 |
| Scrofula, | 9 |

*Diseases of the Ear.*

| | |
|---|---|
| Deafness, | 5 |
| Otorrhœa, | 1 |
| Meatus auditorius wanting, | 1 |

*Diseases of the Organs of Circulation.*

| | |
|---|---|
| Palpitation of heart, | 1 |

*Diseases of the Organs of Respiration.*

| | |
|---|---|
| Chronic Bronchitis, | 1 |

*Diseases of Digestive Organs.*

| | |
|---|---|
| Diarrhœa, | 2 |
| Worms, | 4 |

*Diseases of the Liver.*

| | |
|---|---|
| Chronic ind. and enlargement, | 1 |

*Diseases of the Generative Organs.*

| | |
|---|---|
| Fistula urethræ, | 1 |
| Urinary calculi, | 1 |
| Bubo, | 1 |

| | |
|---|---|
| *Diseases of the Nervous System.* | |
| Paralysis, | 1 |
| *Cutaneous Diseases.* | |
| Tinea capitis, | 4 |
| Scabies, | 3 |
| Lichen circinatus, | 1 |
| Various, | 4 |
| *Diseases of the Bones.* | |
| Osteo-sarcoma, | 1 |
| Hip-joint disease, | 2 |
| Caries of femur, | 1 |
| " of submaxillary, | 1 |
| *Injuries.* | |
| Fracture of radius and ulna, | 1 |
| Disease of chest from bursting of a gun, | 1 |
| Curvature of spine, | 1 |
| Excision of tongue, | 1 |
| Injury from violent exercise, | 1 |
| *Preternatural and Diseased Growths.* | |
| Horny excrescence on head, | 1 |
| Polypi, nasal, | 1 |
| Tumours, sarcomatous, | 2 |
| " cutaneous, | 11 |
| Hydatid of breast, | 1 |
| Scirrhus of breast, | 5 |
| Goitre, | 1 |
| Hypertrophy of the arm, | 1 |
| Atrophy of the arm, | 2 |

### Cases.

*Horn upon the Crown of the Head.*—Chow Keätseuen, aged 31, a florist of Shuntih, had a horn upon his head, just to the right of the "bump of veneration." The patient stated, that some years since he had an encysted tumour upon his head, the integument of which was destroyed by escharotics, and the fluid escaped. The germ of the horn was thus exposed. Its growth had been gradual. Some time previous to coming to the hospital, half an inch or more had been cut off. At this time, the remaining truncated cone was a full inch high, and two inches in circumference at the base. It was of a yellow-white color, and of the usual hardness of horn. It was attached wholly to the integument of the scalp, and gave great pain if pulled.

*Dec. 19th.*—It was removed. Two elliptical incisions were made, so as to take out the whole of the integuments in which it originated. This was preternaturally soft, and the veins and arteries were unusually large and numerous. The wound was brought completely together by sutures and adhesive straps, and in about one week it was quite well.

*Dec. 14th.—Tumour, pendulous from the Upper Lip.*—Kwŏ Pe, aged 27, of Shuntih. Seven years since, this young woman found a tumour commencing on the right side of the upper lip. It had now attained the size of her fist, and hung pendulous, reaching below the chin, and carrying the under lip to the left side; not only disfiguring, but impeding her speech, and requiring to be supported when she eat.

*Dec. 19th.*—The tumour was removed by the hare lip operation; two arteries of considerable size were divided; one needle was introduced, and two or three sutures. A sufficiency of the upper lip was preserved to bring the point of union to the angle of the mouth, so that when united there seemed to have been but one incision from that point, straight to the outer edge of the nose.

*Dec. 22d.*—Third day after the operation, the first time of dressing, the needle was removed. The wound had nearly healed by the first intention, and, on the 5th, only a piece of adhesive plaster was required. In a few days more she was discharged perfectly well, and her natural features nearly restored.

## Figures and Tables

**Figure f1:**